# Effects of energy drinks on mental health and academic performance of university students: A systematic review and meta-analysis protocol

**DOI:** 10.1371/journal.pone.0319533

**Published:** 2025-03-12

**Authors:** Danyla Rafaela Oliveira Batista, Kaio Vinicius C. Silva, Miguel Torres, Woska Pires da Costa, Manuel Monfort-Pañego, Priscilla Rayanne E. Silva, Matias Noll

**Affiliations:** 1 Instituto Federal Goiano - Campus Ceres, Ceres, Goiás, Brazil; 2 Universidade Federal de Goiás (UFG), Goiânia, Goiás, Brazil; 3 Kent Business School, University of Kent, Canterbury, Kent, United Kingdom; 4 Universitat de València, Valencia, Spain; Birjand University of Medical Sciences, IRAN, ISLAMIC REPUBLIC OF

## Abstract

**Objectives:**

Energy drink (ED) consumption is frequently observed among higher education students and is often associated with increased concentration and academic performance. However, the purported benefits are not fully supported by scientific evidence. This protocol details methods for a systematic review and meta-analysis to evaluate the effects of ED on university students’ mental health and academic performance.

**Methods:**

The PECO framework will guide the search strategy, and the protocol will follow the PRISMA-P 2015, PRESS 2015, and PRISMA-S guidelines. The searches will be conducted in the following databases: Web of Science, Scopus, Ovid, PubMed, Embase, FSTA, CINAHL, and SPORTDiscus. The GRADE recommendations and the Downs and Black scale will assess study quality and bias. All statistical analyses will be conducted using Comprehensive Meta-analysis software. We will use Cochran’s *Q* with *p* < 0.05 to indicate significant heterogeneity and *I*² to quantify heterogeneity between studies. The Trim and Fill technique and Egger’s regression test will be applied to evaluate the funnel plot that will be generated.

**Results:**

Carrying out the systematic review proposed by this protocol will provide evidence on the short-, medium-, and long-term effects of ED use on academic performance and mental health among higher education students.

**Conclusion:**

A deeper understanding of ED consumption among higher education students can inform the development of evidence-based educational and public health policies to promote student well-being and mitigate associated health risks.

**Register on PROSPERO:**

CRD42024580044

## Background

Several factors can influence academic performance, including study habits [1] and the consumption of stimulants [2]. Among these, energy drinks (ED) have been proposed to improve this performance [3]. ED contain high levels of substances, such as caffeine, taurine, sugar, and added micronutrients [4,5], known for their energizing and anti-fatigue properties that purportedly enhance concentration and academic performance. However, current evidence supporting these benefits is lacking [6].

Approximately 54.7% of the world’s population has consumed ED at least once, and around 32.3% have consumed it in the past 30 days [7]. Common motivations for ED consumption include staying awake to study [8], staying alert during lessons, improving concentration, meeting assignment deadlines, studying for tests [9], and improving academic and physical performance [10]. However, gaps and inconsistencies in the literature regarding the effects of ED on academic performance remain. While one study suggested that ED improves cognitive performance [11], others linked ED consumption to greater stress [9,12,13], anxiety [12,13], and depression [9,12,13].

The high prevalence of ED consumption and its potential adverse health effects underscore the necessity for further research on this topic [10]. While systematic reviews have explored the effects of ED on mental health in adults [13] and consumption patterns among students [10], none have examined the impact on higher education students’ mental health and academic performance. This lack of integrated evidence leaves open critical questions about the potential benefits and risks of ED consumption in this specific group.

A systematic review in this context would provide robust evidence of the relationship between short-, medium-, and long-term ED consumption and its effects on higher education students’ mental health and academic performance. Therefore, this protocol addresses the question: “What are the effects of ED consumption on the mental health and academic performance of higher education students?”. Here, we aim to analyze the impact of ED consumption on the mental health and academic performance of these students.

## Description of protocol

### Protocol and registration

For the preparation and development of this protocol, we employed the following guidelines: Preferred Reporting Items for Systematic Reviews and Meta-Analyses (PRISMA-P 2015) for reporting the systematic review protocol [14–16], and Peer Review of Electronic Search Strategies (PRESS 2015) to validate the search strategy [17,18]. We will use the PRISMA-S guidelines to document the systematic review conducted based on this protocol [18,19]. The protocol will be registered with PROSPERO to ensure transparency in the proposed study [CRD42024580044] [20]. Any changes made to this protocol during the study will be communicated to PROSPERO [21].

### Search strategy and databases

We used the Population, Exposure, Comparison, and Outcome (PECO) framework to develop a search strategy (Table 1). The PECO framework has the following structure: P =  higher education students, E =  ED consumption, C =  less or no consumption of ED, and O =  mental health and academic performance. Thus, comprehensive database searches will be conducted, including similar terms and synonyms for the terms that constitute the PECO framework (Table 1).

**Table 1:** Search strategy by block.

Search	Entry terms
#1	(student* OR academic* OR graduat* OR undergraduat* OR postgraduat* OR colleg* OR facult* OR universit*)
#2	(“energy drink*” OR “energy beverage*” OR “energy booster*” OR “energy tonic*” OR “high-energy drink*” OR “high-energy beverage*” OR “high-energy booster*” OR “stimulant drink*” OR “stimulant beverage*” OR “stimulant tonic*” OR “performance drink*” OR “performance beverage*” OR “performance booster*” OR “performance tonic*” OR “sports drink*” OR “sports beverage*” OR “sports booster*” OR “sports tonic*” OR “alertness tonic*” OR “revitalizing drink*” OR “revitalizing beverage*” OR “revitalizing tonic*” OR “caffeinated drink*” OR “caffeinated beverage*” OR “caffeinated tonic*” OR “caffeine drink*” OR “caffeine beverage*” OR “caffeine booster*” OR “functional drink*” OR “functional beverage*” OR “functional booster*” OR “functional tonic*” OR “ultra-processed stimulant*” OR “ultra-processed booster*” OR “functional tonic*”)
#3	(“emotional health” OR “emotional disorder” OR “emotional disturbance” OR “emotional problem” OR “emotional well-being” OR “mental health” OR “mental disorder” OR “mental disturbance” OR “mental problem” OR “mental illness” OR “psychological health” OR “psychological disorder” OR “psychological disturbance” OR “psychological problem” OR “psychological well-being” OR “psychiatric health” OR “psychiatric disorder” OR “psychiatric disturbance” OR “psychiatric problem” OR “psychiatric illness” OR depression OR anxiety OR “academic performance” OR “academic achievement” OR “academic success” OR “academic failure” OR “academic progress” OR “academic growth” OR “academic attainment” OR “academic result” OR “academic development” OR “educational performance” OR “educational achievement” OR “educational success” OR “educational failure” OR “educational progress” OR “educational growth” OR “educational attainment” OR “educational result” OR “educational development” OR “learning outcomes” OR “student performance” OR “student achievement” OR “student success” OR “student failure” OR “student progress” OR “student growth” OR “student attainment” OR “student result” OR “student development” OR “learning performance” OR “learning achievement” OR “learning success” OR “learning failure” OR “learning progress” OR “learning growth” OR “learning attainment” OR “learning result” OR “learning development”)
#4	(#1) AND (#2) AND (#3)

Systematic searches and the collection of metadata related to the studies will be carried out in the following databases: Web of Science^™^ Core Collection, Scopus^™^, Ovid^®^, MEDLINE/PubMed^®^, Embase^™^, FSTA via EBSCOhost, Cumulative Index to Nursing and Allied Health Literature (CINAHL^®^) via EBSCOhost, and SPORTDiscus via EBSCOhost. The search string will include the title, abstract, and keywords (when possible) to ensure the inclusion of relevant studies in the analysis [22]. This systematic search constitutes the first phase of data collection.

In the second phase of the search for evidence, we will use the articles included in the first phase of the systematic review. The strategy adopted at this stage will use the Litmaps^®^ platform (https://www.litmaps.com/), which integrates artificial intelligence technology to find similar studies related to an article provided as a reference. For each included article, a query will be made on Litmaps^®^ using the digital object identifier (DOI). All the returned studies will follow the steps adopted in the first phase. The platform uses artificial intelligence to map scientific evidence by cross-referencing and citing citations. The Litmaps^®^ tool will also consult each article included through mapping until no additional eligible articles are found.

### Eligibility criteria

In March 2025, the researcher (R1) will search the databases. The systematic review will identify articles that meet the predefined inclusion criteria without restrictions on language or period of publication, following the recommendations of leading organizations in systematic reviews such as the Cochrane and Campbell Collaboration. The Scite tool (https://scite.ai/) will be used to check for retractions. If retraction records are found, we will delete the articles. Checking for retractions is indispensable because retracted articles contain serious errors or false information that can lead to erroneous conclusions in further research [23]. The eligibility criteria are as follows:

Inclusion criteria:

(i1) Original published and peer-reviewed studies [20,24].
(i2) Studies without language or publication date restrictions.
(i3) Quantitative studies: cross-sectional, cohort, case-control, and controlled clinical trials [25].
(i4) Studies with an adult population (higher education students).
(i5) Studies that evaluated the relationships between energy drink consumption, mental health, and/or academic performance.

Exclusion criteria:

(e1) Duplicates: If multiple articles were published by the same author on the same dataset and topic, only the most comprehensive article would be considered [22]. Duplicates will be removed following Bramer’s method [26], and a manual review will be conducted to confirm their exclusion [20,27].
(e2) Studies that evaluated the consumption of ED with alcohol.
(e3) Studies that were not fully available in the searched databases and could not be accessed even after attempts to contact the authors [20,24].
(e4) Studies with data from different population profiles that could not be separated from the target group of higher education students.
(e5) Studies that considered higher education students with physical and mental disabilities [20].
(e6) Studies with a retraction record up to the point before submission of the systematic review to a scientific journal [18].

If no study meets the inclusion criteria, the systematic review will be classified as an empty review [28].

### Review process

The following steps will be taken in the selection process:

i) Deduplication process: The articles found in the database search will be imported into Rayyan^®^ (available at https://www.rayyan.ai/) to remove duplications automatically.
ii) Screening process: Two reviewers (R1 and R2) will select studies based on the eligibility criteria by reading the titles and abstracts of the identified studies on Rayyan^®^, maintaining blinding between the reviewers.
iii) The concordance ratio and Cohen’s kappa coefficient will be calculated to measure reviewer agreement.
iv) Disagreements between reviewers: Disagreements will be resolved by a third senior reviewer (R3) based on previously established eligibility criteria.
v) Complete reading of the study: Once the screening is completed, the selected articles will be read in full to confirm their eligibility for inclusion.

**Fig 1:**
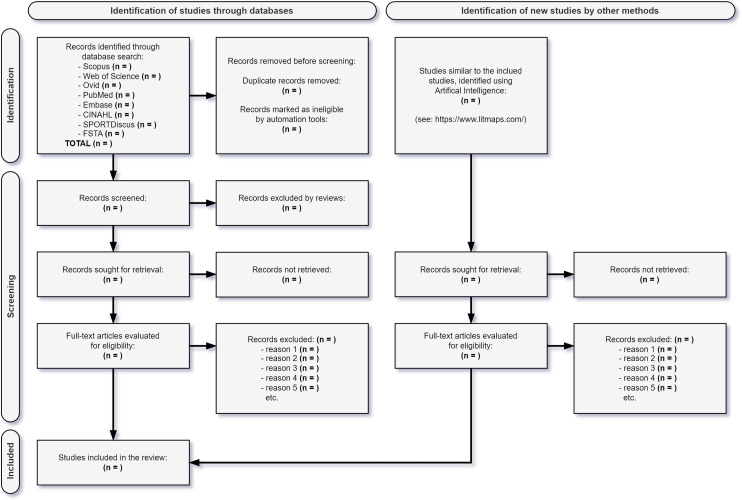
PRISMA 2020 flow diagram for identifying, screening, and including studies in the review.

The systematic review will follow the PRISMA 2020 guidelines according to the flowchart of the study selection process [29] (Fig 1).

### Reviewers’ training

We will train two reviewers (R1 and R2) to select articles using the eligibility criteria with 50 titles and abstracts before coding them [30–32]. For this training, we will use Rayyan^®^ software. This software enhances the efficiency of selecting studies for systematic reviews [33] using artificial intelligence to learn from user decisions and build a model for predicting article relevance throughout the screening process [22].

Finally, we will conduct a training session for all project participants to ensure a consistent understanding and standardize the assessment of the risk of bias and data extraction from the articles [30,32]. This training will evaluate five studies not included in the final review, ensuring that all participants are fully prepared for the systematic review process.

### Assessment of risk of bias

Two reviewers (R1 and R2) will independently assess the risk of bias. In the event of a disagreement between the two reviewers, a third independent reviewer (R3) will be consulted to resolve the disagreement [34]. The risk of bias in quantitative studies will be assessed using the Downs and Black scale [35,36]. The quality indicator of each selected study will be calculated as a score expressed in percentage format [34] and categorized as “low risk of bias” (≥70%) or “high risk of bias” (<70%) [36,37].

### Data extraction and synthesis

Data will be extracted and evaluated by two independent reviewers (R1 and R2), and any disagreements will be resolved by a third senior reviewer (R3) [22]. If necessary, one of the researchers will contact the authors to obtain relevant missing data related to the included studies [34]. Data from these studies will be recorded in a standardized spreadsheet and summarized in tables and graphs containing the following information: 1) title (DOI), 2) author’s name and year of publication, 3) nationality, 4) type of study, 5) age, 6) sample size, 7) sex, 8) type of energy drink, and 9) outcome.

The characteristics of the included studies will be summarized using descriptive statistics. We will present the frequencies for categorical data (e.g., related to the meta-analysis approach and risk of bias). We will report numerical data as means, standard deviations, medians, and interquartile ranges. If possible, we will simplify the data (e.g., pooling means and standard deviations) to better describe the population of each result.

### Data analysis

All the statistical analyses will be performed via Comprehensive Meta-Analysis software version 4.0 for Microsoft Windows^™^ (Biostat, Inc., Englewood, NJ). The summarized effect will be determined by considering the random-effects model, and the effects will be summarized using their 95% confidence intervals. In addition to *Q,* with *p* < 0.05 indicating significant heterogeneity, we will also conduct the *I*² statistic to quantify the heterogeneity between studies. Heterogeneity can be classified as low (0–25%), moderate (25–50%), or high (>50%) [38].

Subgroup analyses will investigate the effect of categorical variables, such as age groups, gender, and context variables, on study outcomes. To complement these analyses, meta-regressions will be performed to evaluate the effect of continuous variables, including demographic factors and the duration of energy drink usage, on the effect size [39]. Separate meta-analyses will be conducted for each type of study to ensure methodological rigor.

Meta-analyses rely on the data and effect sizes reported in the included studies. Whenever adjusted effect sizes are available, they will be prioritized in the analysis, as these account for confounding factors. Studies that only report crude effect sizes will be used to ensure data inclusion.

To perform sensitivity analyses, we will consider the impact (1) of each study individually and (2) variability in study quality [39]. First, we will conduct several analyses that exclude one study at a time to determine if any specific study significantly alters the effect size [39]. Additionally, we will conduct further analyses by stratifying the studies based on their risk of bias (i.e., low, moderate, and high risk of bias). This approach will allow us to assess the consistency of effect sizes across studies of varying quality, leading to a more robust interpretation of the results [39].

A funnel plot will be generated to evaluate publication bias, which shows the dispersion of effect sizes relative to their standard errors across studies [40]. However, following the recommendations of the Cochrane Handbook, funnel plots will only be plotted when there are at least 10 studies in the meta-analysis, as the power of statistical tests and visual interpretations is insufficient for smaller datasets [41]. For datasets with fewer than 10 studies, Begg’s Rank Correlation Test will be used to assess publication bias, as the statistical power of tests like Egger’s regression test can be limited in small datasets [42].

For datasets with 10 or more studies, Egger’s regression test will be employed [43], as it is more sensitive and effective in detecting publication bias in larger datasets [42]. Egger’s regression test will use a significance threshold of *p* < 0.05 for detecting publication bias. The Trim and Fill method will be applied to adjust the results for potentially missing studies [40], estimate the corrected effect size, and address potential asymmetry in the funnel plot [42]. To complement these analyses, Rosenthal’s fail-safe N method will be used as an additional approach to evaluate the robustness of the findings by estimating the number of missing studies required to render the results statistically nonsignificant [42]. As the visual inspection of this method is subjective, we will also use the Trim and Fill technique [40] and Egger’s regression test [44].

The results will be categorized [22,45] and may be presented using figures, diagrams, or other graphic elements for better visualization [46,47] and understanding [48]. We will use forest plots to present effect size estimates and their corresponding 95% confidence intervals across individual studies. Bubble plots will illustrate relationships between moderators and outcomes for meta-regressions.

After analyzing and interpreting the results, we will submit a systematic review to a peer-reviewed scientific journal. The extracted metadata (raw data) will be published as supplementary material to ensure the transparency of the study and to make it possible to reproduce the results. In addition, we will consider making the metadata available on an open-access platform.

### Certainty of evidence

The summarized results will be evaluated based on the certainty of the evidence using the Grading of Recommendations, Assessment, Development, and Evaluations (GRADE) recommendations [49–51]. The certainty ratings of the evidence can be “high,” “moderate,” “low,” or “very low” [52]. The results of observational studies will be classified as evidence of low certainty; however, the certainty can be increased or reduced according to other evaluations [52]. This change will be based on the following five factors:

- Risk of bias (downward adjustment by one level if 50–75% of the studies are at “low risk” of bias and by two if it is < 50%).
- Consistency (downward adjustment by one level for “moderate heterogeneity” and by two for “high heterogeneity”).
- Precision (downward adjustment by one level if the power is 80–90% and by two if it is < 80%).
- Generalizing results (downward adjustment by one level if generalized to different populations).
- Publication bias (downward adjustment by one level if there was publication bias according to Egger’s test).

In the case of significant and very-large effects, the certainty will be increased by one and two levels, respectively [52]. In this context, if the summarized effect of the prevalence ratio (derived from meta-analyses of cross-sectional studies) or the relative risk (derived from meta-analyses of cohort studies) is less than 0.5 or greater than 2, the level of certainty in the evidence will be increased by one level. If the ratio is less than 0.2 or greater than 5, the level of certainty will be increased by two levels [53].

## Results

Through analysis of existing research, this systematic review will provide evidence on ED use’s short-, medium-, and long-term effects on academic performance and mental health among higher education students. We hope the findings will generate recommendations for future research and inform public health policies regarding ED use among higher education students.

## Discussion

This protocol aims to analyze the impact of ED consumption on the mental health and academic performance of higher education students. Previous analyses indicate statistically significant differences in lifetime consumption prevalence rates between continents (p = 0.035) [7]. North America has the highest prevalence (62.1%), followed by Europe (55.5%), Asia (49.1%), Oceania (48.3%), and Central and South America (39.1%) [7].

Globally, concerns about ED consumption have resulted in regulatory proposals focused on labeling the amount of caffeine, limiting its content, and restricting advertising [54]. In 2014, Lithuania banned the sale of EDs to people under the age of 18 [55]. In 2018, several UK supermarket chains stopped selling energy drinks to customers under 16 years [54]. The European Union requires energy drinks to have a “high caffeine content” label [55]. Regional variations in ED consumption can be attributed to sociocultural, economic, and regulatory factors.

Existing research suggests both positive and negative effects of ED, but it is still unclear whether they are more beneficial or harmful. Previous studies have focused on other populations and outcomes and have overlooked essential assessments such as risk of bias, publication bias, and certainty of evidence. To date, we have not identified any systematic reviews that explore the effects of ED on the academic performance of higher education students using a more robust meta-analytic approach. One systematic review, for example, investigated the impact of these drinks on mental health [13], but the authors did not adopt a meta-analytical approach and worked with a heterogeneous population [13]. Another study, which examined the effects of ED in a pediatric population [56], did not analyze their relationship with stress and depression.

Assessing the risk of publication bias is essential for determining the certainty of the evidence [57]. However, none of these studies included this analysis. Thus, a comprehensive systematic review focused on higher education students is urgently needed to provide evidence-based insights into ED effects on academic performance and mental health, filling existing gaps in the literature and guiding future research and policy development.

If the link between excessive consumption of ED, mental health problems, and reduced academic performance is proven, it becomes essential to implement educational and regulatory measures. This includes warnings on labels and information actions in educational institutions. Policies adapted to regional specificities – considering cultural and socio-economic aspects – combined with interventions in the academic environment, such as re-evaluating deadlines and greater flexibility for working students, can reduce dependence on these drinks and promote a healthier and more balanced learning environment.

### Limitations and strengths

This study has limitations and strengths that deserve consideration. Among the limitations, confounding factors, such as lifestyle habits (diet, sleep, physical activity), substance use, and academic stress, stand out, which can impact the results regardless of ED consumption, making it difficult to attribute specific effects to these beverages.

The exclusion of qualitative studies is also a limitation, as it prevents the capture of nuances related to students’ consumption contexts and motivations. The heterogeneity of the designs, populations, ED, and outcomes assessed can make comparing and generalizing the results difficult. We will address these limitations using a subgroup meta-analysis [39]. In addition, we will evaluate the risk of bias ratio and strength of evidence for each summarized outcome, which will consider heterogeneities [39].

While individual participant data meta-analyses could allow for more detailed subgroup analyses and exploration of contextual and demographic factors, logistical and ethical challenges make such an approach impractical for the current study. Therefore, we will perform subgroup analyses using the aggregate data available, focusing on key variables such as age, gender, and socioeconomic status where reported. Although these analyses are less detailed than individual participant data meta-analyses, they still provide valuable insights into potential differences across subpopulations [39].

Among the strengths are the use of several databases, the non-restriction of language and date of publication, and the use of the Litmaps^®^ tool to identify similar studies. The study follows well-established frameworks, including the PECO framework for structuring research questions, and the PRISMA-P, PRESS, and PRISMA-S guidelines, as well as registration in PROSPERO. At the same time carefully assessing the risk of bias, publication bias, and certainty of evidence lends greater rigor to the study. Thus, the resulting meta-analyses are expected to provide a solid and reliable quantitative synthesis.

## Conclusion

This protocol addresses the growing concern about ED consumption among higher education students by providing robust evidence of its relationship with physical and mental health and academic performance. By enhancing our understanding of these relationships, our findings will inform educational and public health policies that promote student well-being. Implementing these policies can improve higher education students’ academic environment.

## Supporting information

S1 File The PRISMA-P checklist for this systematic review protocol. (DOCX)

S2 File Details of the Boolean search string for each database. (DOCX)
