# Supplementary material for: Effects of energy drinks on mental health and academic performance of university students: A systematic review and meta-analysis protocol
Source: PLoS One. 2025 Mar 12;20(3):e0319533. doi: 10.1371/journal.pone.0319533 (PMC11902126; doi:10.1371/journal.pone.0319533)
Supplement: S2 File — (DOCX) [file pone.0319533.s002.docx]

**Search strategy tables**

| **Scopus** | |
| --- | --- |
| **#1** | TITLE-ABS-KEY((student* OR academic* OR graduat* OR undergraduat* OR postgraduat* OR colleg* OR facult* OR universit*)) |
| **#2** | TITLE-ABS-KEY(("energy drink*" OR "energy beverage*" OR "energy booster*" OR "energy tonic*" OR "high-energy drink*" OR "high-energy beverage*" OR "high-energy booster*" OR "stimulant drink*" OR "stimulant beverage*" OR "stimulant tonic*" OR "performance drink*" OR "performance beverage*" OR "performance booster*" OR "performance tonic*" OR "sports drink*" OR "sports beverage*" OR "sports booster*" OR "sports tonic*" OR "alertness tonic*" OR "revitalizing drink*" OR "revitalizing beverage*" OR "revitalizing tonic*" OR "caffeinated drink*" OR "caffeinated beverage*" OR "caffeinated tonic*" OR "caffeine drink*" OR "caffeine beverage*" OR "caffeine booster*" OR "functional drink*" OR "functional beverage*" OR "functional booster*" OR "functional tonic*" OR "ultra-processed stimulant*" OR "ultra-processed booster*" OR "functional tonic*")) |
| **#3** | TITLE-ABS-KEY(("emotional health" OR "emotional disorder" OR "emotional disturbance" OR "emotional problem" OR "emotional well-being" OR "mental health" OR "mental disorder" OR "mental disturbance" OR "mental problem" OR "mental illness" OR "psychological health" OR "psychological disorder" OR "psychological disturbance" OR "psychological problem" OR "psychological well-being" OR "psychiatric health" OR "psychiatric disorder" OR "psychiatric disturbance" OR "psychiatric problem" OR "psychiatric illness" OR depression OR anxiety OR "academic performance" OR "academic achievement" OR "academic success" OR "academic failure" OR "academic progress" OR "academic growth" OR "academic attainment" OR "academic result" OR "academic development" OR "educational performance" OR "educational achievement" OR "educational success" OR "educational failure" OR "educational progress" OR "educational growth" OR "educational attainment" OR "educational result" OR "educational development" OR "learning outcomes" OR "student performance" OR "student achievement" OR "student success" OR "student failure" OR "student progress" OR "student growth" OR "student attainment" OR "student result" OR "student development" OR "learning performance" OR "learning achievement" OR "learning success" OR "learning failure" OR "learning progress" OR "learning growth" OR "learning attainment" OR "learning result" OR "learning development")) |
| **#4** | (#1) AND (#2) AND (#3) |

| **Web of Science** | |
| --- | --- |
| **#1** | TS=((student* OR academic* OR graduat* OR undergraduat* OR postgraduat* OR colleg* OR facult* OR universit*)) |
| **#2** | TS=(("energy drink*" OR "energy beverage*" OR "energy booster*" OR "energy tonic*" OR "high-energy drink*" OR "high-energy beverage*" OR "high-energy booster*" OR "stimulant drink*" OR "stimulant beverage*" OR "stimulant tonic*" OR "performance drink*" OR "performance beverage*" OR "performance booster*" OR "performance tonic*" OR "sports drink*" OR "sports beverage*" OR "sports booster*" OR "sports tonic*" OR "alertness tonic*" OR "revitalizing drink*" OR "revitalizing beverage*" OR "revitalizing tonic*" OR "caffeinated drink*" OR "caffeinated beverage*" OR "caffeinated tonic*" OR "caffeine drink*" OR "caffeine beverage*" OR "caffeine booster*" OR "functional drink*" OR "functional beverage*" OR "functional booster*" OR "functional tonic*" OR "ultra-processed stimulant*" OR "ultra-processed booster*" OR "functional tonic*")) |
| **#3** | TS=(("emotional health" OR "emotional disorder" OR "emotional disturbance" OR "emotional problem" OR "emotional well-being" OR "mental health" OR "mental disorder" OR "mental disturbance" OR "mental problem" OR "mental illness" OR "psychological health" OR "psychological disorder" OR "psychological disturbance" OR "psychological problem" OR "psychological well-being" OR "psychiatric health" OR "psychiatric disorder" OR "psychiatric disturbance" OR "psychiatric problem" OR "psychiatric illness" OR depression OR anxiety OR "academic performance" OR "academic achievement" OR "academic success" OR "academic failure" OR "academic progress" OR "academic growth" OR "academic attainment" OR "academic result" OR "academic development" OR "educational performance" OR "educational achievement" OR "educational success" OR "educational failure" OR "educational progress" OR "educational growth" OR "educational attainment" OR "educational result" OR "educational development" OR "learning outcomes" OR "student performance" OR "student achievement" OR "student success" OR "student failure" OR "student progress" OR "student growth" OR "student attainment" OR "student result" OR "student development" OR "learning performance" OR "learning achievement" OR "learning success" OR "learning failure" OR "learning progress" OR "learning growth" OR "learning attainment" OR "learning result" OR "learning development")) |
| **#4** | (#1) AND (#2) AND (#3) |

| **Ovid** | |
| --- | --- |
| **#1** | (student* or academic* or graduat* or undergraduat* or postgraduat* or colleg* or facult* or universit*).ti. or (student* or academic* or graduat* or undergraduat* or postgraduat* or colleg* or facult* or universit*).ab. or (student* or academic* or graduat* or undergraduat* or postgraduat* or colleg* or facult* or universit*).kw. |
| **#2** | ("energy drink*" or "energy beverage*" or "energy booster*" or "energy tonic*" or "high-energy drink*" or "high-energy beverage*" or "high-energy booster*" or "stimulant drink*" or "stimulant beverage*" or "stimulant tonic*" or "performance drink*" or "performance beverage*" or "performance booster*" or "performance tonic*" or "sports drink*" or "sports beverage*" or "sports booster*" or "sports tonic*" or "alertness tonic*" or "revitalizing drink*" or "revitalizing beverage*" or "revitalizing tonic*" or "caffeinated drink*" or "caffeinated beverage*" or "caffeinated tonic*" or "caffeine drink*" or "caffeine beverage*" or "caffeine booster*" or "functional drink*" or "functional beverage*" or "functional booster*" or "functional tonic*" or "ultra-processed stimulant*" or "ultra-processed booster*" or "functional tonic*").ti. or ("energy drink*" or "energy beverage*" or "energy booster*" or "energy tonic*" or "high-energy drink*" or "high-energy beverage*" or "high-energy booster*" or "stimulant drink*" or "stimulant beverage*" or "stimulant tonic*" or "performance drink*" or "performance beverage*" or "performance booster*" or "performance tonic*" or "sports drink*" or "sports beverage*" or "sports booster*" or "sports tonic*" or "alertness tonic*" or "revitalizing drink*" or "revitalizing beverage*" or "revitalizing tonic*" or "caffeinated drink*" or "caffeinated beverage*" or "caffeinated tonic*" or "caffeine drink*" or "caffeine beverage*" or "caffeine booster*" or "functional drink*" or "functional beverage*" or "functional booster*" or "functional tonic*" or "ultra-processed stimulant*" or "ultra-processed booster*" or "functional tonic*").ab. or ("energy drink*" or "energy beverage*" or "energy booster*" or "energy tonic*" or "high-energy drink*" or "high-energy beverage*" or "high-energy booster*" or "stimulant drink*" or "stimulant beverage*" or "stimulant tonic*" or "performance drink*" or "performance beverage*" or "performance booster*" or "performance tonic*" or "sports drink*" or "sports beverage*" or "sports booster*" or "sports tonic*" or "alertness tonic*" or "revitalizing drink*" or "revitalizing beverage*" or "revitalizing tonic*" or "caffeinated drink*" or "caffeinated beverage*" or "caffeinated tonic*" or "caffeine drink*" or "caffeine beverage*" or "caffeine booster*" or "functional drink*" or "functional beverage*" or "functional booster*" or "functional tonic*" or "ultra-processed stimulant*" or "ultra-processed booster*" or "functional tonic*").kw. |
| **#3** | ("emotional health" or "emotional disorder" or "emotional disturbance" or "emotional problem" or "emotional well-being" or "mental health" or "mental disorder" or "mental disturbance" or "mental problem" or "mental illness" or "psychological health" or "psychological disorder" or "psychological disturbance" or "psychological problem" or "psychological well-being" or "psychiatric health" or "psychiatric disorder" or "psychiatric disturbance" or "psychiatric problem" or "psychiatric illness" or depression or anxiety or "academic performance" or "academic achievement" or "academic success" or "academic failure" or "academic progress" or "academic growth" or "academic attainment" or "academic result" or "academic development" or "educational performance" or "educational achievement" or "educational success" or "educational failure" or "educational progress" or "educational growth" or "educational attainment" or "educational result" or "educational development" or "learning outcomes" or "student performance" or "student achievement" or "student success" or "student failure" or "student progress" or "student growth" or "student attainment" or "student result" or "student development" or "learning performance" or "learning achievement" or "learning success" or "learning failure" or "learning progress" or "learning growth" or "learning attainment" or "learning result" or "learning development").ti. or ("emotional health" or "emotional disorder" or "emotional disturbance" or "emotional problem" or "emotional well-being" or "mental health" or "mental disorder" or "mental disturbance" or "mental problem" or "mental illness" or "psychological health" or "psychological disorder" or "psychological disturbance" or "psychological problem" or "psychological well-being" or "psychiatric health" or "psychiatric disorder" or "psychiatric disturbance" or "psychiatric problem" or "psychiatric illness" or depression or anxiety or "academic performance" or "academic achievement" or "academic success" or "academic failure" or "academic progress" or "academic growth" or "academic attainment" or "academic result" or "academic development" or "educational performance" or "educational achievement" or "educational success" or "educational failure" or "educational progress" or "educational growth" or "educational attainment" or "educational result" or "educational development" or "learning outcomes" or "student performance" or "student achievement" or "student success" or "student failure" or "student progress" or "student growth" or "student attainment" or "student result" or "student development" or "learning performance" or "learning achievement" or "learning success" or "learning failure" or "learning progress" or "learning growth" or "learning attainment" or "learning result" or "learning development").ab. or ("emotional health" or "emotional disorder" or "emotional disturbance" or "emotional problem" or "emotional well-being" or "mental health" or "mental disorder" or "mental disturbance" or "mental problem" or "mental illness" or "psychological health" or "psychological disorder" or "psychological disturbance" or "psychological problem" or "psychological well-being" or "psychiatric health" or "psychiatric disorder" or "psychiatric disturbance" or "psychiatric problem" or "psychiatric illness" or depression or anxiety or "academic performance" or "academic achievement" or "academic success" or "academic failure" or "academic progress" or "academic growth" or "academic attainment" or "academic result" or "academic development" or "educational performance" or "educational achievement" or "educational success" or "educational failure" or "educational progress" or "educational growth" or "educational attainment" or "educational result" or "educational development" or "learning outcomes" or "student performance" or "student achievement" or "student success" or "student failure" or "student progress" or "student growth" or "student attainment" or "student result" or "student development" or "learning performance" or "learning achievement" or "learning success" or "learning failure" or "learning progress" or "learning growth" or "learning attainment" or "learning result" or "learning development").kw. |
| **#4** | (#1) AND (#2) AND (#3) |

| **Embase** | |
| --- | --- |
| **#1** | (student*:ti,ab,kw OR academic*:ti,ab,kw OR graduat*:ti,ab,kw OR undergraduat*:ti,ab,kw OR postgraduat*:ti,ab,kw OR colleg*:ti,ab,kw OR facult*:ti,ab,kw OR universit*:ti,ab,kw) |
| **#2** | ('energy drink*':ti,ab,kw OR 'energy beverage*':ti,ab,kw OR 'energy booster*':ti,ab,kw OR 'energy tonic*':ti,ab,kw OR 'high-energy drink*':ti,ab,kw OR 'high-energy beverage*':ti,ab,kw OR 'high-energy booster*':ti,ab,kw OR 'stimulant drink*':ti,ab,kw OR 'stimulant beverage*':ti,ab,kw OR 'stimulant tonic*':ti,ab,kw OR 'performance drink*':ti,ab,kw OR 'performance beverage*':ti,ab,kw OR 'performance booster*':ti,ab,kw OR 'performance tonic*':ti,ab,kw OR 'sports drink*':ti,ab,kw OR 'sports beverage*':ti,ab,kw OR 'sports booster*':ti,ab,kw OR 'sports tonic*':ti,ab,kw OR 'alertness tonic*':ti,ab,kw OR 'revitalizing drink*':ti,ab,kw OR 'revitalizing beverage*':ti,ab,kw OR 'revitalizing tonic*':ti,ab,kw OR 'caffeinated drink*':ti,ab,kw OR 'caffeinated beverage*':ti,ab,kw OR 'caffeinated tonic*':ti,ab,kw OR 'caffeine drink*':ti,ab,kw OR 'caffeine beverage*':ti,ab,kw OR 'caffeine booster*':ti,ab,kw OR 'functional drink*':ti,ab,kw OR 'functional beverage*':ti,ab,kw OR 'functional booster*':ti,ab,kw OR 'ultra-processed stimulant*':ti,ab,kw OR 'ultra-processed booster*':ti,ab,kw OR 'functional tonic*':ti,ab,kw) |
| **#3** | ('emotional health':ti,ab,kw OR 'emotional disorder':ti,ab,kw OR 'emotional disturbance':ti,ab,kw OR 'emotional problem':ti,ab,kw OR 'emotional well-being':ti,ab,kw OR 'mental health':ti,ab,kw OR 'mental disorder':ti,ab,kw OR 'mental disturbance':ti,ab,kw OR 'mental problem':ti,ab,kw OR 'mental illness':ti,ab,kw OR 'psychological health':ti,ab,kw OR 'psychological disorder':ti,ab,kw OR 'psychological disturbance':ti,ab,kw OR 'psychological problem':ti,ab,kw OR 'psychological well-being':ti,ab,kw OR 'psychiatric health':ti,ab,kw OR 'psychiatric disorder':ti,ab,kw OR 'psychiatric disturbance':ti,ab,kw OR 'psychiatric problem':ti,ab,kw OR 'psychiatric illness':ti,ab,kw OR depression:ti,ab,kw OR anxiety:ti,ab,kw OR 'academic performance':ti,ab,kw OR 'academic achievement':ti,ab,kw OR 'academic success':ti,ab,kw OR 'academic failure':ti,ab,kw OR 'academic progress':ti,ab,kw OR 'academic growth':ti,ab,kw OR 'academic attainment':ti,ab,kw OR 'academic result':ti,ab,kw OR 'academic development':ti,ab,kw OR 'educational performance':ti,ab,kw OR 'educational achievement':ti,ab,kw OR 'educational success':ti,ab,kw OR 'educational failure':ti,ab,kw OR 'educational progress':ti,ab,kw OR 'educational growth':ti,ab,kw OR 'educational attainment':ti,ab,kw OR 'educational result':ti,ab,kw OR 'educational development':ti,ab,kw OR 'learning outcomes':ti,ab,kw OR 'student performance':ti,ab,kw OR 'student achievement':ti,ab,kw OR 'student success':ti,ab,kw OR 'student failure':ti,ab,kw OR 'student progress':ti,ab,kw OR 'student growth':ti,ab,kw OR 'student attainment':ti,ab,kw OR 'student result':ti,ab,kw OR 'student development':ti,ab,kw OR 'learning performance':ti,ab,kw OR 'learning achievement':ti,ab,kw OR 'learning success':ti,ab,kw OR 'learning failure':ti,ab,kw OR 'learning progress':ti,ab,kw OR 'learning growth':ti,ab,kw OR 'learning attainment':ti,ab,kw OR 'learning result':ti,ab,kw OR 'learning development':ti,ab,kw) |
| **#4** | (#1) AND (#2) AND (#3) |

| **FSTA** | |
| --- | --- |
| **#1** | TI ( (student* OR academic* OR graduat* OR undergraduat* OR postgraduat* OR colleg* OR facult* OR universit*) ) OR AB ( (student* OR academic* OR graduat* OR undergraduat* OR postgraduat* OR colleg* OR facult* OR universit*) ) OR KW ( (student* OR academic* OR graduat* OR undergraduat* OR postgraduat* OR colleg* OR facult* OR universit*) ) |
| **#2** | TI ( ("energy drink*" OR "energy beverage*" OR "energy booster*" OR "energy tonic*" OR "high-energy drink*" OR "high-energy beverage*" OR "high-energy booster*" OR "stimulant drink*" OR "stimulant beverage*" OR "stimulant tonic*" OR "performance drink*" OR "performance beverage*" OR "performance booster*" OR "performance tonic*" OR "sports drink*" OR "sports beverage*" OR "sports booster*" OR "sports tonic*" OR "alertness tonic*" OR "revitalizing drink*" OR "revitalizing beverage*" OR "revitalizing tonic*" OR "caffeinated drink*" OR "caffeinated beverage*" OR "caffeinated tonic*" OR "caffeine drink*" OR "caffeine beverage*" OR "caffeine booster*" OR "functional drink*" OR "functional beverage*" OR "functional booster*" OR "functional tonic*" OR "ultra-processed stimulant*" OR "ultra-processed booster*" OR "functional tonic*") ) OR AB ( ("energy drink*" OR "energy beverage*" OR "energy booster*" OR "energy tonic*" OR "high-energy drink*" OR "high-energy beverage*" OR "high-energy booster*" OR "stimulant drink*" OR "stimulant beverage*" OR "stimulant tonic*" OR "performance drink*" OR "performance beverage*" OR "performance booster*" OR "performance tonic*" OR "sports drink*" OR "sports beverage*" OR "sports booster*" OR "sports tonic*" OR "alertness tonic*" OR "revitalizing drink*" OR "revitalizing beverage*" OR "revitalizing tonic*" OR "caffeinated drink*" OR "caffeinated beverage*" OR "caffeinated tonic*" OR "caffeine drink*" OR "caffeine beverage*" OR "caffeine booster*" OR "functional drink*" OR "functional beverage*" OR "functional booster*" OR "functional tonic*" OR "ultra-processed stimulant*" OR "ultra-processed booster*" OR "functional tonic*") ) OR KW ( ("energy drink*" OR "energy beverage*" OR "energy booster*" OR "energy tonic*" OR "high-energy drink*" OR "high-energy beverage*" OR "high-energy booster*" OR "stimulant drink*" OR "stimulant beverage*" OR "stimulant tonic*" OR "performance drink*" OR "performance beverage*" OR "performance booster*" OR "performance tonic*" OR "sports drink*" OR "sports beverage*" OR "sports booster*" OR "sports tonic*" OR "alertness tonic*" OR "revitalizing drink*" OR "revitalizing beverage*" OR "revitalizing tonic*" OR "caffeinated drink*" OR "caffeinated beverage*" OR "caffeinated tonic*" OR "caffeine drink*" OR "caffeine beverage*" OR "caffeine booster*" OR "functional drink*" OR "functional beverage*" OR "functional booster*" OR "functional tonic*" OR "ultra-processed stimulant*" OR "ultra-processed booster*" OR "functional tonic*") ) |
| **#3** | TI ( ("emotional health" OR "emotional disorder" OR "emotional disturbance" OR "emotional problem" OR "emotional well-being" OR "mental health" OR "mental disorder" OR "mental disturbance" OR "mental problem" OR "mental illness" OR "psychological health" OR "psychological disorder" OR "psychological disturbance" OR "psychological problem" OR "psychological well-being" OR "psychiatric health" OR "psychiatric disorder" OR "psychiatric disturbance" OR "psychiatric problem" OR "psychiatric illness" OR depression OR anxiety OR "academic performance" OR "academic achievement" OR "academic success" OR "academic failure" OR "academic progress" OR "academic growth" OR "academic attainment" OR "academic result" OR "academic development" OR "educational performance" OR "educational achievement" OR "educational success" OR "educational failure" OR "educational progress" OR "educational growth" OR "educational attainment" OR "educational result" OR "educational development" OR "learning outcomes" OR "student performance" OR "student achievement" OR "student success" OR "student failure" OR "student progress" OR "student growth" OR "student attainment" OR "student result" OR "student development" OR "learning performance" OR "learning achievement" OR "learning success" OR "learning failure" OR "learning progress" OR "learning growth" OR "learning attainment" OR "learning result" OR "learning development") ) OR AB ( ("emotional health" OR "emotional disorder" OR "emotional disturbance" OR "emotional problem" OR "emotional well-being" OR "mental health" OR "mental disorder" OR "mental disturbance" OR "mental problem" OR "mental illness" OR "psychological health" OR "psychological disorder" OR "psychological disturbance" OR "psychological problem" OR "psychological well-being" OR "psychiatric health" OR "psychiatric disorder" OR "psychiatric disturbance" OR "psychiatric problem" OR "psychiatric illness" OR depression OR anxiety OR "academic performance" OR "academic achievement" OR "academic success" OR "academic failure" OR "academic progress" OR "academic growth" OR "academic attainment" OR "academic result" OR "academic development" OR "educational performance" OR "educational achievement" OR "educational success" OR "educational failure" OR "educational progress" OR "educational growth" OR "educational attainment" OR "educational result" OR "educational development" OR "learning outcomes" OR "student performance" OR "student achievement" OR "student success" OR "student failure" OR "student progress" OR "student growth" OR "student attainment" OR "student result" OR "student development" OR "learning performance" OR "learning achievement" OR "learning success" OR "learning failure" OR "learning progress" OR "learning growth" OR "learning attainment" OR "learning result" OR "learning development") ) OR KW ( ("emotional health" OR "emotional disorder" OR "emotional disturbance" OR "emotional problem" OR "emotional well-being" OR "mental health" OR "mental disorder" OR "mental disturbance" OR "mental problem" OR "mental illness" OR "psychological health" OR "psychological disorder" OR "psychological disturbance" OR "psychological problem" OR "psychological well-being" OR "psychiatric health" OR "psychiatric disorder" OR "psychiatric disturbance" OR "psychiatric problem" OR "psychiatric illness" OR depression OR anxiety OR "academic performance" OR "academic achievement" OR "academic success" OR "academic failure" OR "academic progress" OR "academic growth" OR "academic attainment" OR "academic result" OR "academic development" OR "educational performance" OR "educational achievement" OR "educational success" OR "educational failure" OR "educational progress" OR "educational growth" OR "educational attainment" OR "educational result" OR "educational development" OR "learning outcomes" OR "student performance" OR "student achievement" OR "student success" OR "student failure" OR "student progress" OR "student growth" OR "student attainment" OR "student result" OR "student development" OR "learning performance" OR "learning achievement" OR "learning success" OR "learning failure" OR "learning progress" OR "learning growth" OR "learning attainment" OR "learning result" OR "learning development") ) |
| **#4** | (#1) AND (#2) AND (#3) |

| **CINAHL** | |
| --- | --- |
| **#1** | TI ( student* OR academic* OR graduat* OR undergraduat* OR postgraduat* OR colleg* OR facult* OR universit* ) OR AB ( student* OR academic* OR graduat* OR undergraduat* OR postgraduat* OR colleg* OR facult* OR universit* ) OR MW ( student* OR academic* OR graduat* OR undergraduat* OR postgraduat* OR colleg* OR facult* OR universit* ) |
| **#2** | TI ( ("energy drink*" OR "energy beverage*" OR "energy booster*" OR "energy tonic*" OR "high-energy drink*" OR "high-energy beverage*" OR "high-energy booster*" OR "stimulant drink*" OR "stimulant beverage*" OR "stimulant tonic*" OR "performance drink*" OR "performance beverage*" OR "performance booster*" OR "performance tonic*" OR "sports drink*" OR "sports beverage*" OR "sports booster*" OR "sports tonic*" OR "alertness tonic*" OR "revitalizing drink*" OR "revitalizing beverage*" OR "revitalizing tonic*" OR "caffeinated drink*" OR "caffeinated beverage*" OR "caffeinated tonic*" OR "caffeine drink*" OR "caffeine beverage*" OR "caffeine booster*" OR "functional drink*" OR "functional beverage*" OR "functional booster*" OR "functional tonic*" OR "ultra-processed stimulant*" OR "ultra-processed booster*" OR "functional tonic*") ) OR AB ( ("energy drink*" OR "energy beverage*" OR "energy booster*" OR "energy tonic*" OR "high-energy drink*" OR "high-energy beverage*" OR "high-energy booster*" OR "stimulant drink*" OR "stimulant beverage*" OR "stimulant tonic*" OR "performance drink*" OR "performance beverage*" OR "performance booster*" OR "performance tonic*" OR "sports drink*" OR "sports beverage*" OR "sports booster*" OR "sports tonic*" OR "alertness tonic*" OR "revitalizing drink*" OR "revitalizing beverage*" OR "revitalizing tonic*" OR "caffeinated drink*" OR "caffeinated beverage*" OR "caffeinated tonic*" OR "caffeine drink*" OR "caffeine beverage*" OR "caffeine booster*" OR "functional drink*" OR "functional beverage*" OR "functional booster*" OR "functional tonic*" OR "ultra-processed stimulant*" OR "ultra-processed booster*" OR "functional tonic*") ) OR MW ( ("energy drink*" OR "energy beverage*" OR "energy booster*" OR "energy tonic*" OR "high-energy drink*" OR "high-energy beverage*" OR "high-energy booster*" OR "stimulant drink*" OR "stimulant beverage*" OR "stimulant tonic*" OR "performance drink*" OR "performance beverage*" OR "performance booster*" OR "performance tonic*" OR "sports drink*" OR "sports beverage*" OR "sports booster*" OR "sports tonic*" OR "alertness tonic*" OR "revitalizing drink*" OR "revitalizing beverage*" OR "revitalizing tonic*" OR "caffeinated drink*" OR "caffeinated beverage*" OR "caffeinated tonic*" OR "caffeine drink*" OR "caffeine beverage*" OR "caffeine booster*" OR "functional drink*" OR "functional beverage*" OR "functional booster*" OR "functional tonic*" OR "ultra-processed stimulant*" OR "ultra-processed booster*" OR "functional tonic*") ) |
| **#3** | TI ( "emotional health" OR "emotional disorder" OR "emotional disturbance" OR "emotional problem" OR "emotional well-being" OR "mental health" OR "mental disorder" OR "mental disturbance" OR "mental problem" OR "mental illness" OR "psychological health" OR "psychological disorder" OR "psychological disturbance" OR "psychological problem" OR "psychological well-being" OR "psychiatric health" OR "psychiatric disorder" OR "psychiatric disturbance" OR "psychiatric problem" OR "psychiatric illness" OR depression OR anxiety OR "academic performance" OR "academic achievement" OR "academic success" OR "academic failure" OR "academic progress" OR "academic growth" OR "academic attainment" OR "academic result" OR "academic development" OR "educational performance" OR "educational achievement" OR "educational success" OR "educational failure" OR "educational progress" OR "educational growth" OR "educational attainment" OR "educational result" OR "educational development" OR "learning outcomes" OR "student performance" OR "student achievement" OR "student success" OR "student failure" OR "student progress" OR "student growth" OR "student attainment" OR "student result" OR "student development" OR "learning performance" OR "learning achievement" OR "learning success" OR "learning failure" OR "learning progress" OR "learning growth" OR "learning attainment" OR "learning result" OR "learning development" ) OR AB ( "emotional health" OR "emotional disorder" OR "emotional disturbance" OR "emotional problem" OR "emotional well-being" OR "mental health" OR "mental disorder" OR "mental disturbance" OR "mental problem" OR "mental illness" OR "psychological health" OR "psychological disorder" OR "psychological disturbance" OR "psychological problem" OR "psychological well-being" OR "psychiatric health" OR "psychiatric disorder" OR "psychiatric disturbance" OR "psychiatric problem" OR "psychiatric illness" OR depression OR anxiety OR "academic performance" OR "academic achievement" OR "academic success" OR "academic failure" OR "academic progress" OR "academic growth" OR "academic attainment" OR "academic result" OR "academic development" OR "educational performance" OR "educational achievement" OR "educational success" OR "educational failure" OR "educational progress" OR "educational growth" OR "educational attainment" OR "educational result" OR "educational development" OR "learning outcomes" OR "student performance" OR "student achievement" OR "student success" OR "student failure" OR "student progress" OR "student growth" OR "student attainment" OR "student result" OR "student development" OR "learning performance" OR "learning achievement" OR "learning success" OR "learning failure" OR "learning progress" OR "learning growth" OR "learning attainment" OR "learning result" OR "learning development" ) OR MW ( "emotional health" OR "emotional disorder" OR "emotional disturbance" OR "emotional problem" OR "emotional well-being" OR "mental health" OR "mental disorder" OR "mental disturbance" OR "mental problem" OR "mental illness" OR "psychological health" OR "psychological disorder" OR "psychological disturbance" OR "psychological problem" OR "psychological well-being" OR "psychiatric health" OR "psychiatric disorder" OR "psychiatric disturbance" OR "psychiatric problem" OR "psychiatric illness" OR depression OR anxiety OR "academic performance" OR "academic achievement" OR "academic success" OR "academic failure" OR "academic progress" OR "academic growth" OR "academic attainment" OR "academic result" OR "academic development" OR "educational performance" OR "educational achievement" OR "educational success" OR "educational failure" OR "educational progress" OR "educational growth" OR "educational attainment" OR "educational result" OR "educational development" OR "learning outcomes" OR "student performance" OR "student achievement" OR "student success" OR "student failure" OR "student progress" OR "student growth" OR "student attainment" OR "student result" OR "student development" OR "learning performance" OR "learning achievement" OR "learning success" OR "learning failure" OR "learning progress" OR "learning growth" OR "learning attainment" OR "learning result" OR "learning development" ) |
| **#4** | (#1) AND (#2) AND (#3) |

| **SPORTDiscus** | |
| --- | --- |
| **#1** | TI ( (student* OR academic* OR graduat* OR undergraduat* OR postgraduat* OR colleg* OR facult* OR universit*) ) OR AB ( (student* OR academic* OR graduat* OR undergraduat* OR postgraduat* OR colleg* OR facult* OR universit*) ) OR KW ( (student* OR academic* OR graduat* OR undergraduat* OR postgraduat* OR colleg* OR facult* OR universit*) ) |
| **#2** | ("energy drink*" OR "energy beverage*" OR "energy booster*" OR "energy tonic*" OR "high-energy drink*" OR "high-energy beverage*" OR "high-energy booster*" OR "stimulant drink*" OR "stimulant beverage*" OR "stimulant tonic*" OR "performance drink*" OR "performance beverage*" OR "performance booster*" OR "performance tonic*" OR "sports drink*" OR "sports beverage*" OR "sports booster*" OR "sports tonic*" OR "alertness tonic*" OR "revitalizing drink*" OR "revitalizing beverage*" OR "revitalizing tonic*" OR "caffeinated drink*" OR "caffeinated beverage*" OR "caffeinated tonic*" OR "caffeine drink*" OR "caffeine beverage*" OR "caffeine booster*" OR "functional drink*" OR "functional beverage*" OR "functional booster*" OR "functional tonic*" OR "ultra-processed stimulant*" OR "ultra-processed booster*" OR "functional tonic*") |
| **#3** | TI ( ("emotional health" OR "emotional disorder" OR "emotional disturbance" OR "emotional problem" OR "emotional well-being" OR "mental health" OR "mental disorder" OR "mental disturbance" OR "mental problem" OR "mental illness" OR "psychological health" OR "psychological disorder" OR "psychological disturbance" OR "psychological problem" OR "psychological well-being" OR "psychiatric health" OR "psychiatric disorder" OR "psychiatric disturbance" OR "psychiatric problem" OR "psychiatric illness" OR depression OR anxiety OR "academic performance" OR "academic achievement" OR "academic success" OR "academic failure" OR "academic progress" OR "academic growth" OR "academic attainment" OR "academic result" OR "academic development" OR "educational performance" OR "educational achievement" OR "educational success" OR "educational failure" OR "educational progress" OR "educational growth" OR "educational attainment" OR "educational result" OR "educational development" OR "learning outcomes" OR "student performance" OR "student achievement" OR "student success" OR "student failure" OR "student progress" OR "student growth" OR "student attainment" OR "student result" OR "student development" OR "learning performance" OR "learning achievement" OR "learning success" OR "learning failure" OR "learning progress" OR "learning growth" OR "learning attainment" OR "learning result" OR "learning development") ) OR AB ( ("emotional health" OR "emotional disorder" OR "emotional disturbance" OR "emotional problem" OR "emotional well-being" OR "mental health" OR "mental disorder" OR "mental disturbance" OR "mental problem" OR "mental illness" OR "psychological health" OR "psychological disorder" OR "psychological disturbance" OR "psychological problem" OR "psychological well-being" OR "psychiatric health" OR "psychiatric disorder" OR "psychiatric disturbance" OR "psychiatric problem" OR "psychiatric illness" OR depression OR anxiety OR "academic performance" OR "academic achievement" OR "academic success" OR "academic failure" OR "academic progress" OR "academic growth" OR "academic attainment" OR "academic result" OR "academic development" OR "educational performance" OR "educational achievement" OR "educational success" OR "educational failure" OR "educational progress" OR "educational growth" OR "educational attainment" OR "educational result" OR "educational development" OR "learning outcomes" OR "student performance" OR "student achievement" OR "student success" OR "student failure" OR "student progress" OR "student growth" OR "student attainment" OR "student result" OR "student development" OR "learning performance" OR "learning achievement" OR "learning success" OR "learning failure" OR "learning progress" OR "learning growth" OR "learning attainment" OR "learning result" OR "learning development") ) OR KW ( ("emotional health" OR "emotional disorder" OR "emotional disturbance" OR "emotional problem" OR "emotional well-being" OR "mental health" OR "mental disorder" OR "mental disturbance" OR "mental problem" OR "mental illness" OR "psychological health" OR "psychological disorder" OR "psychological disturbance" OR "psychological problem" OR "psychological well-being" OR "psychiatric health" OR "psychiatric disorder" OR "psychiatric disturbance" OR "psychiatric problem" OR "psychiatric illness" OR depression OR anxiety OR "academic performance" OR "academic achievement" OR "academic success" OR "academic failure" OR "academic progress" OR "academic growth" OR "academic attainment" OR "academic result" OR "academic development" OR "educational performance" OR "educational achievement" OR "educational success" OR "educational failure" OR "educational progress" OR "educational growth" OR "educational attainment" OR "educational result" OR "educational development" OR "learning outcomes" OR "student performance" OR "student achievement" OR "student success" OR "student failure" OR "student progress" OR "student growth" OR "student attainment" OR "student result" OR "student development" OR "learning performance" OR "learning achievement" OR "learning success" OR "learning failure" OR "learning progress" OR "learning growth" OR "learning attainment" OR "learning result" OR "learning development") |
| **#4** | (#1) AND (#2) AND (#3) |

| **PubMed** | |
| --- | --- |
| **#1** | (student*[Title/Abstract] OR academic*[Title/Abstract] OR graduat*[Title/Abstract] OR undergraduat*[Title/Abstract] OR postgraduat*[Title/Abstract] OR colleg*[Title/Abstract] OR facult*[Title/Abstract] OR universit*[Title/Abstract] OR "students"[MeSH Terms] OR "academia"[MeSH Terms] OR "faculty"[MeSH Terms] OR "universities"[MeSH Terms]) |
| **#2** | (energy drink*[Title/Abstract] OR energy beverage*[Title/Abstract] OR energy booster*[Title/Abstract] OR energy tonic*[Title/Abstract] OR high-energy drink*[Title/Abstract] OR high-energy beverage*[Title/Abstract] OR high-energy booster*[Title/Abstract] OR stimulant drink*[Title/Abstract] OR stimulant beverage*[Title/Abstract] OR stimulant tonic*[Title/Abstract] OR performance drink*[Title/Abstract] OR performance beverage*[Title/Abstract] OR performance booster*[Title/Abstract] OR performance tonic*[Title/Abstract] OR sports drink*[Title/Abstract] OR sports beverage*[Title/Abstract] OR sports booster*[Title/Abstract] OR sports tonic*[Title/Abstract] OR alertness tonic*[Title/Abstract] OR revitalizing drink*[Title/Abstract] OR revitalizing beverage*[Title/Abstract] OR revitalizing tonic*[Title/Abstract] OR caffeinated drink*[Title/Abstract] OR caffeinated beverage*[Title/Abstract] OR caffeinated tonic*[Title/Abstract] OR caffeine drink*[Title/Abstract] OR caffeine beverage*[Title/Abstract] OR caffeine booster*[Title/Abstract] OR functional drink*[Title/Abstract] OR functional beverage*[Title/Abstract] OR functional booster*[Title/Abstract] OR functional tonic*[Title/Abstract] OR ultra-processed stimulant*[Title/Abstract] OR ultra-processed booster*[Title/Abstract] OR functional tonic*[Title/Abstract] OR energy drinks[MeSH Terms]) |
| **#3** | ("emotional health"[Title/Abstract] OR "emotional disorder"[Title/Abstract] OR "emotional disturbance"[Title/Abstract] OR "emotional problem"[Title/Abstract] OR "emotional well-being"[Title/Abstract] OR "mental health"[Title/Abstract] OR "mental disorder"[Title/Abstract] OR "mental disturbance"[Title/Abstract] OR "mental problem"[Title/Abstract] OR "mental illness"[Title/Abstract] OR "psychological health"[Title/Abstract] OR "psychological disorder"[Title/Abstract] OR "psychological disturbance"[Title/Abstract] OR "psychological problem"[Title/Abstract] OR "psychological well-being"[Title/Abstract] OR "psychiatric health"[Title/Abstract] OR "psychiatric disorder"[Title/Abstract] OR "psychiatric disturbance"[Title/Abstract] OR "psychiatric problem"[Title/Abstract] OR "psychiatric illness"[Title/Abstract] OR depression[Title/Abstract] OR anxiety[Title/Abstract] OR "academic performance"[Title/Abstract] OR "academic achievement"[Title/Abstract] OR "academic success"[Title/Abstract] OR "academic failure"[Title/Abstract] OR "academic progress"[Title/Abstract] OR "academic growth"[Title/Abstract] OR "academic attainment"[Title/Abstract] OR "academic result"[Title/Abstract] OR "academic development"[Title/Abstract] OR "educational performance"[Title/Abstract] OR "educational achievement"[Title/Abstract] OR "educational success"[Title/Abstract] OR "educational failure"[Title/Abstract] OR "educational progress"[Title/Abstract] OR "educational growth"[Title/Abstract] OR "educational attainment"[Title/Abstract] OR "educational result"[Title/Abstract] OR "educational development"[Title/Abstract] OR "learning outcomes"[Title/Abstract] OR "student performance"[Title/Abstract] OR "student achievement"[Title/Abstract] OR "student success"[Title/Abstract] OR "student failure"[Title/Abstract] OR "student progress"[Title/Abstract] OR "student growth"[Title/Abstract] OR "student attainment"[Title/Abstract] OR "student result"[Title/Abstract] OR "student development"[Title/Abstract] OR "learning performance"[Title/Abstract] OR "learning achievement"[Title/Abstract] OR "learning success"[Title/Abstract] OR "learning failure"[Title/Abstract] OR "learning progress"[Title/Abstract] OR "learning growth"[Title/Abstract] OR "learning attainment"[Title/Abstract] OR "learning result"[Title/Abstract] OR "learning development"[Title/Abstract] OR "mental disorders"[MeSH Terms] OR "depressive disorder"[MeSH Terms] OR "depressive disorder"[MeSH Terms] OR "depression"[MeSH Terms] OR "anxiety"[MeSH Terms] OR "academic performance"[MeSH Terms]) |
| **#4** | (#1) AND (#2) AND (#3) |
